# Efficacy of surgical treatment on polypharmacy of elderly patients with lumbar spinal canal stenosis: retrospective exploratory research

**DOI:** 10.1186/s12877-023-03853-x

**Published:** 2023-03-24

**Authors:** Sota Nagai, Risa Inagaki, Takehiro Michikawa, Soya Kawabata, Kaori Ito, Kurenai Hachiya, Hiroki Takeda, Daiki Ikeda, Shinjiro Kaneko, Shigeki Yamada, Nobuyuki Fujita

**Affiliations:** 1 Department of Orthopaedic Surgery, School of Medicine, Fujita Health University, 1-98 Dengakugakubo, Kutsukake-cho, Toyoake, Aichi 470-1192 Japan; 2 Department of Clinical Pharmacy, School of Medicine, Fujita Health University, Aichi, Japan; 3 Department of Environmental and Occupational Health, School of Medicine, Toho University, Tokyo, Japan; 4 Department of Hematology, School of Medicine, Fujita Health University, Aichi, Japan; 5 Department of Spine and Spinal Cord Surgery, School of Medicine, Fujita Health University, Aichi, Japan

**Keywords:** Polypharmacy, Lumbar spinal canal stenosis, Lumbar spinal surgery, Elderly patients, Psychological condition

## Abstract

**Background:**

Polypharmacy is a growing public health problem occurring in all healthcare settings worldwide. Elderly patients with lumbar spinal canal stenosis (LSS) who manifest low back and neuropathic pain and have a high frequency of comorbidity are predicted to take many drugs. However, no studies have reported polypharmacy in elderly patients with LSS. Thus, we aimed to review the polypharmacy among elderly LSS patients with elective surgeries and examine how the surgical treatment reduces the polypharmacy.

**Methods:**

We retrospectively enrolled all the patients aged ≥ 65 years who underwent spinal surgery for LSS between April 2020 and March 2021. The prescribed drugs of participants were directly checked by pharmacists in the outpatient department preoperatively and 6-month and 1-year postoperatively. The baseline characteristics were collected beside the patient-based outcomes including Roland–Morris Disability Questionnaire, Zurich Claudication Questionnaire, and Japanese Orthopaedic Association Back Pain Evaluation Questionnaire (JOABPEQ). The cutoff number of drugs for polypharmacy was defined as 6. The prescription drugs were divided into 9 categories: drugs for neuropsychiatric, cardiovascular, respiratory, digestive, endocrine metabolic, and urinary renal diseases; blood products; pain relief medication; and others.

**Results:**

A total of 102 cases were finally analyzed, with a follow-up rate of 78.0%. Of the participants, the preoperative polypharmacy prevalence was 66.7%. The number of drugs 6-month and 1-year postoperatively was significantly less than the preoperative one. The proportions of polypharmacy at 6 months and 1 year after surgery significantly decreased to 57.8% and 55.9%, respectively. When the prescribed drugs were divided into 9 categories, the number of drugs for pain relief and digestive diseases was significantly reduced after surgery. The multi-variable analysis revealed that a higher score in the psychological disorder of JOABPEQ was associated with 3 or more drugs decreased 1-year postoperatively (OR, 2.5; 95% CI: 1.0–6.1).

**Conclusion:**

Polypharmacy prevalence was high among elderly LSS patients indicated for lumbar spinal surgery. Additionally, our data showed that lumbar spinal surgery was effective in reducing polypharmacy among elderly LSS patients. Finally, the multi-variable analysis indicated that better psychological condition was associated with the reduction of prescribed drugs after lumbar spinal surgery.

## Background

As the world moves toward an aging society, the proportion growth of the elderly is a global health and socioeconomic problem [1]. Due to population growth and aging, the number of patients with musculoskeletal disorders is increasing rapidly [1, 2]. Lumbar spinal canal stenosis (LSS), one of the common degenerative musculoskeletal disorders, is caused by a narrowing of the lumbar spinal canal that compresses the cauda equina and nerve roots. In the computed tomography study using a US community-based sample, LSS prevalence was about 19% among patients in the 60s [3]. A Japanese population-based study using a self-administered questionnaire for LSS reported that the prevalence was around 11% in the 70s, which increased with age [4]. The first-line treatment for LSS is conservative therapy, such as pharmacotherapy, exercise therapy, and block therapy [5]. There are many drug options for LSS, including Non-Steroidal Anti-Inflammatory Drugs (NSAIDs), opioids, serotonin-noradrenalin reuptake inhibitors (SNRIs), pregabalin/mirogabalin, prostaglandin E1 analogs (PGE1), acetaminophen, and so on [6]. On the other hand, surgical treatment, including decompression and/or fusion, is indicated for LSS patients who are refractory to conservative therapy, and the outcomes are generally favorable [7, 8].

Polypharmacy, taking multiple drugs simultaneously, is common among elderlies because they usually suffer from numerous diseases. The cutoff number for drugs in polypharmacy is not clearly defined, but commonly 5 or 6 [9, 10]. Polypharmacy is associated with the use of potentially inappropriate medications [9], resulting in increased adverse drug events (ADEs), including poor treatment adherence, reduction of physical function, decreased cognitive function, and high risk of falls [11–15]. WHO reported that polypharmacy is a growing public health problem occurring in all healthcare settings worldwide [16]. A recent study reported that degenerative lumbar spinal disorders including LSS were significantly associated with polypharmacy in elderlies with degenerative musculoskeletal disorders [17]. In particular, LSS patients with neuropathic pain and multiple comorbidities are prone to get more drugs [18, 19]. In addition, since LSS patients were at high risk of falls due to decreased motor function of the lower extremities [20], it is essential to prevent polypharmacy associated with risk of falls. However, to the best of our knowledge, no studies have reported polypharmacy in elderly patients with LSS. Additionally, the effect of surgical treatment for LSS patients on polypharmacy is also unclear. Therefore, the first aim of this study was to investigate the detail of drugs prescribed to elderly patients with elective surgeries for LSS. The second was to examine how the surgical treatment for LSS reduces the number of prescribed drugs.

## Methods

### Study participants

The research design of this study was a retrospective observational study. We retrospectively enrolled all the patients aged ≥ 65 years who underwent lumbar spinal surgery for LSS at our institution between April 2020 and March 2021. The follow-up period was one year. Surgical treatment was indicated for patients with clear LSS symptoms, including leg pain and numbness and neurogenic claudication, and who were refractory to conservative therapy according to the guideline [5]. Since this was an exploratory analysis of observational study, we did not estimate sample size and continued to include participants throughout the duration of the study. LSS diagnosis was confirmed using MRI, myelography, or computed tomography. Cases with fusion segments ≥ 4 were excluded before enrollment.We defined spondylolisthesis as an anterior slip of the upper vertebra ≥ 5% and degenerative lumbar scoliosis as Cobb angle ≥ 10°. Cases with multiple spinal lumbar surgeries were defined as failed back surgery syndrome (FBSS). Posterior fusion was recommended for patients with spondylolisthesis and/or a posterior opening > 5° on dynamic lateral radiographs. Case with additional lumbar surgery during the follow-up period was excluded.

### Ethics approval

The ethics committee of our institution granted ethical approval for this study (approval No. HM20-530). The ethics committee approved the inclusion of all eligible patients in the study unless we were contacted to opt-out. All study methods were conducted in accordance with the guidelines set out in the Declaration of Helsinki.

### Data collection

Prescribed drugs of participants were directly checked by pharmacists in the outpatient department preoperatively and 6-month and 1-year postoperatively. The participants have the same physician who prescribe the drugs before and after surgery. The drug was prescribed at the discretion of the individual physician. The prescribing physician was unaware that the patient was a participant in this study. The participants with 6 medications were considered to have polypharmacy. Enrolled patients were assessed preoperatively in addition to 6-month and 1-year after the surgery for the patient-based outcomes including Roland–Morris Disability Questionnaire (RDQ), Zurich Claudication Questionnaire (ZCQ), and Japanese Orthopaedic Association Back Pain Evaluation Questionnaire (JOABPEQ). We collected the following data for each patient: age; gender; body mass index (BMI); medical history, including type 2 diabetes mellitus (DM), hypertension, hyperlipidemia, cardiovascular disease, cerebrovascular disease, and cancer; spondylolisthesis; degenerative lumbar scoliosis; FBSS; perioperative factors including surgical procedure such as decompression and/or fusion, the number of decompression level, surgical time, and surgical blood loss. The surgical treatment was considered “effective” or “not effective” according to the JOABPEQ based on the following; an increase of ≥ 20 points in the postoperative score over the preoperative one, or a preoperative score < 90 with a postoperative score ≥ 90 points [20].

### Classification of the drugs

According to the previous study [17], the prescription drugs were divided into 9 categories: drugs for neuropsychiatric, cardiovascular, respiratory, digestive, endocrine metabolic, and urinary renal diseases; blood products; pain relief medication; and others. Antithrombotic drugs were included in the category of drugs for cardiovascular diseases. Osteoporosis drugs were included in the category of drugs for endocrine metabolic diseases. Pain relief medication included NSAIDs, pregabalin/mirogabalin, opioids, SNRIs, and acetaminophen.

### Statistical analyses

The data among groups were compared using Chi-squared test, McNemar’s test, or Wilcoxon signed-rank test, as appropriate. P values < 0.05 were considered to indicate statistical significance.When we performed the McNamer and Wilcoxon signed rank test more than once, a P value of 0.025 (0.05/2) was used as a statistically significance to avoid the type 1 error. To examine the independent associations of 1-year postoperative decrease of 3 or more drugs, we constructed a Poisson regression model that included age, sex, metabolic component, FBSS, surgical procedure, and preoperative score in each domain of JOABPEQ, and estimated relative risk (RR) and 95% confidence intervals (CIs) for 1-year postoperative decrease of 3 or more drugs. In the Poisson regression model, JOABPEQ was selected as an explanatory variable among the three patient-based outcomes because JOABPEQ can be divided into five domains: pain disorder, lumbar function, walking ability, social life, and psychological disorder, and patients’ conditions can be evaluated in detail [21]. Because there is no clinical cutoff value for categorizing the scores in each domain of JOABPEQ, the scores of JOABPEQ were categorized in tertiles to account for the number of participants. In addition, because scores of JOABEPQ have misclassification, we divided them into groups with similar scores. The metabolic component was defined as having at least one of the following; BMI of 25 or higher, DM, hypertension, and hyperlipidemia [22]. Poisson regression was performed using the STATA16 software (Stata Corporation, College Station, TX, USA).

## Results

**Fig. 1:**
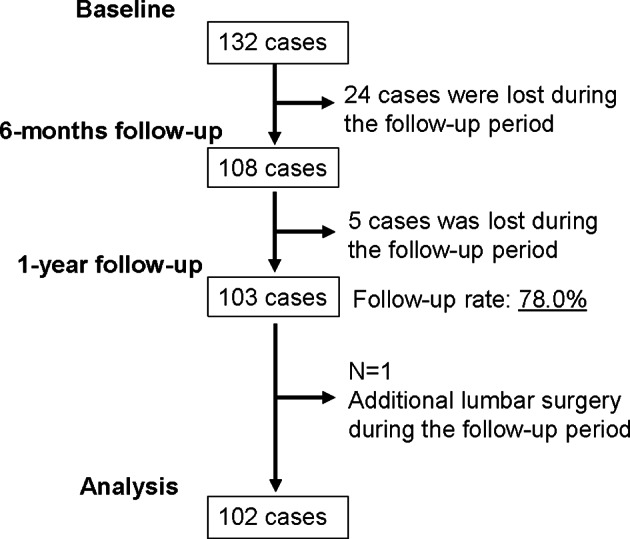
Flow diagram showing the flow of participants through each stage in this study

Totally, 132 patients were enrolled in this study. A total of 29 cases were lost during the follow-up period, with a follow-up rate of 78.0% (Fig. 1). One case with additional lumbar surgery during the follow-up period were excluded (Fig. 1). Finally, a total of 102 cases were analyzed. Table 1 summarizes baseline characteristics. Table 2 shows the preoperative score of RDQ, ZCQ including symptom severity and physical function, and JOABPEQ, in addition to the data for 6-month and 1-year postoperatively. Postoperative RDQ, ZCQ, and JOABPEQ scores (6-month and 1-year) were significantly improved compared to the preoperative data. The frequency of “effective” in each domain of JOABPEQ was also favorable.

**Table 1:** Baseline Characteristics

Patients		n = 102
Gender		Male: 56 Female: 46
Age (yeas)		75.5 ± 5.9
BMI (kg/m2)		23.8 ± 3.4
Medical history	Diabetes mellitus	31 (30.4%)
	Hypertension	65 (63.7%)
	Hyperlipidemia	56 (55.0%)
	Cardiovascular disease	34 (33.3%)
	Cerebrovascular disease	11 (10.8%)
	Cancer	18 (17.6%)
Duration of conservative therapy before surgery	< 6 months	28 (27.5%)
	> = 6 months, < 1 year	17 (16.7%)
	> = 1 year, < 3 year	31 (30.4%)
	> = 3 years	26 (24.5%)
Spondylolisthesis		35 (34.3%)
Degenerative lumbar scoloiosis		14 (13.7%)
FBSS		8 (7.8%)
Surgical procedure	decompression	59 (57.8%)
	decompression + fusion	43 (42.2%)
Surgical time (min)		140.1 ± 90.5
Surgical blood loss (ml)		150.4 ± 168.2
Decompression levels		2.0 ± 0.9
FBSS, Failed back surgery syndrome		

**Table 2:** Valuables of patient reported outcome at baseline and follow-up after surgery (n = 102)

		Median (25-75%tile)			p value*	
		Preoperation	6POM	1POY	preoperation vs. 6POM	preoperation vs. 1POY
RDQ		13 (7–17)	6 (1–11)	6 (0–12)	< 0.01	< 0.01
ZCQ	Symptom severity	3.3 (2.9–3.7)	2.3 (1.6–2.7)	2.1 (1.7–2.7)	< 0.01	< 0.01
	Physical function	2.8 (2.2–3.2)	1.6 (1.2–2.2)	1.6 (1.2–2.2)	< 0.01	< 0.01
JOABPEQ	Pain disorder	43 (14–71)	100 (71–100)	100 (43–100)	< 0.01	< 0.01
	Lumbar function	50 (25–75)	79 (50–100)	83 (42–83)	< 0.01	< 0.01
	Walking ability	21 (0–43)	68 (36–100)	71 (33–93)	< 0.01	< 0.01
	Social life	35 (22–51)	57 (39–78)	59 (44–81)	< 0.01	< 0.01
	Psychological disorder	44 (32–53)	55 (45–69)	60 (48–72)	< 0.01	< 0.01
The number of effective case of surgical treatment on JOABPEQ	Pain disorder		71(69.6%)	62 (60.8%)		
	Lumbar function		53(52.0%)	54 (52.4%)		
	Walking ability		67(65.7%)	69 (67.0%)		
	Social life		46(45.0%)	59 (57.3%)		
	Psychological disorder		31(30.0%)	35 (34.0%)		

RDQ, Roland–Morris Disability Questionnaire; ZCQ, Zurich Claudication Questionnaire; JOABPEQ, JOA Back Pain Evaluation Questionnaire

*Wilcoxon signed-rank test

**Fig. 2:**
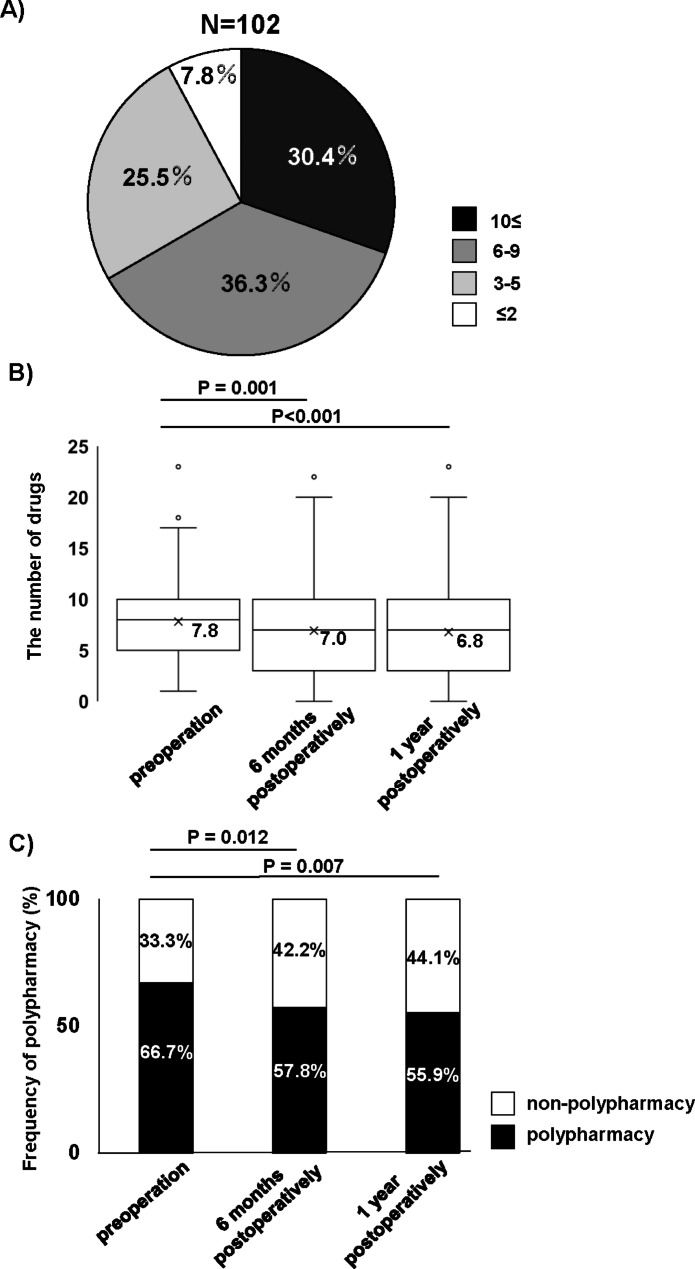
A) The distribution of the number of preoperative prescription drugs in all the participants. B) Comparison of the preoperative and 6-month and 1-year postoperative number of prescribed drugs in the participants. C) Comparison of the preoperative and 6-month and 1-year postoperative proportion of polypharmacy

Figure 2A shows the distribution of the number of preoperative prescribed drugs among the participants. When the cutoff number of drugs was 6, the prevalence of polypharmacy was 66.7%. Figure 2B presents the preoperative and 6-month and 1-year postoperative number of prescribed drugs in the participants. The number of drugs 6-month and 1-year postoperatively were significantly less than the preoperative one. Figure 2C shows the preoperative and 6-month and 1-year postoperative proportion of polypharmacy. The proportions of polypharmacy at 6 months and 1 year after surgery were significantly reduced to 57.8% and 55.9%, respectively.

**Fig. 3:**
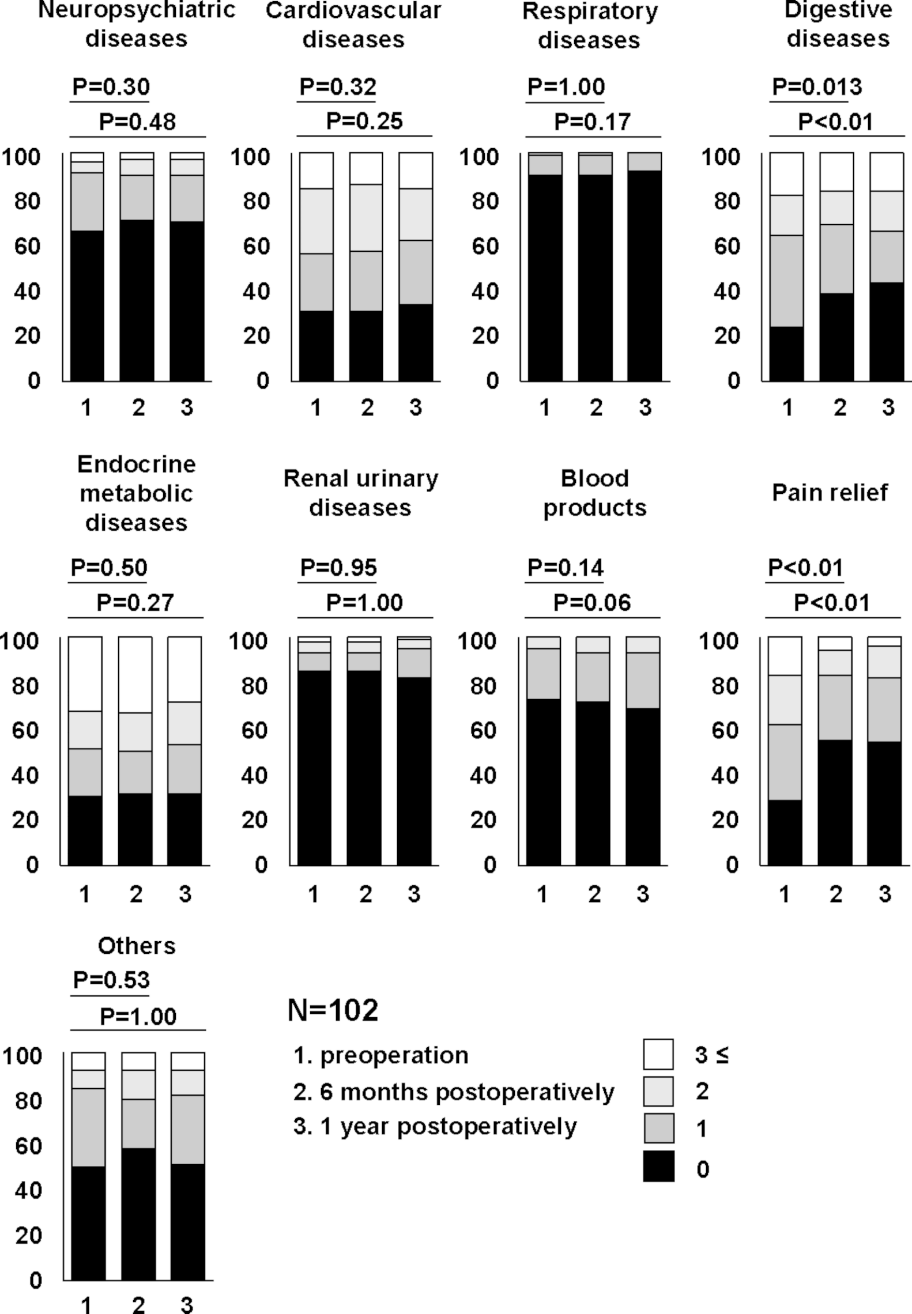
Comparison of the preoperative and 6-month and 1-year postoperative number of prescribed drugs in 9 categories

**Fig. 4:**
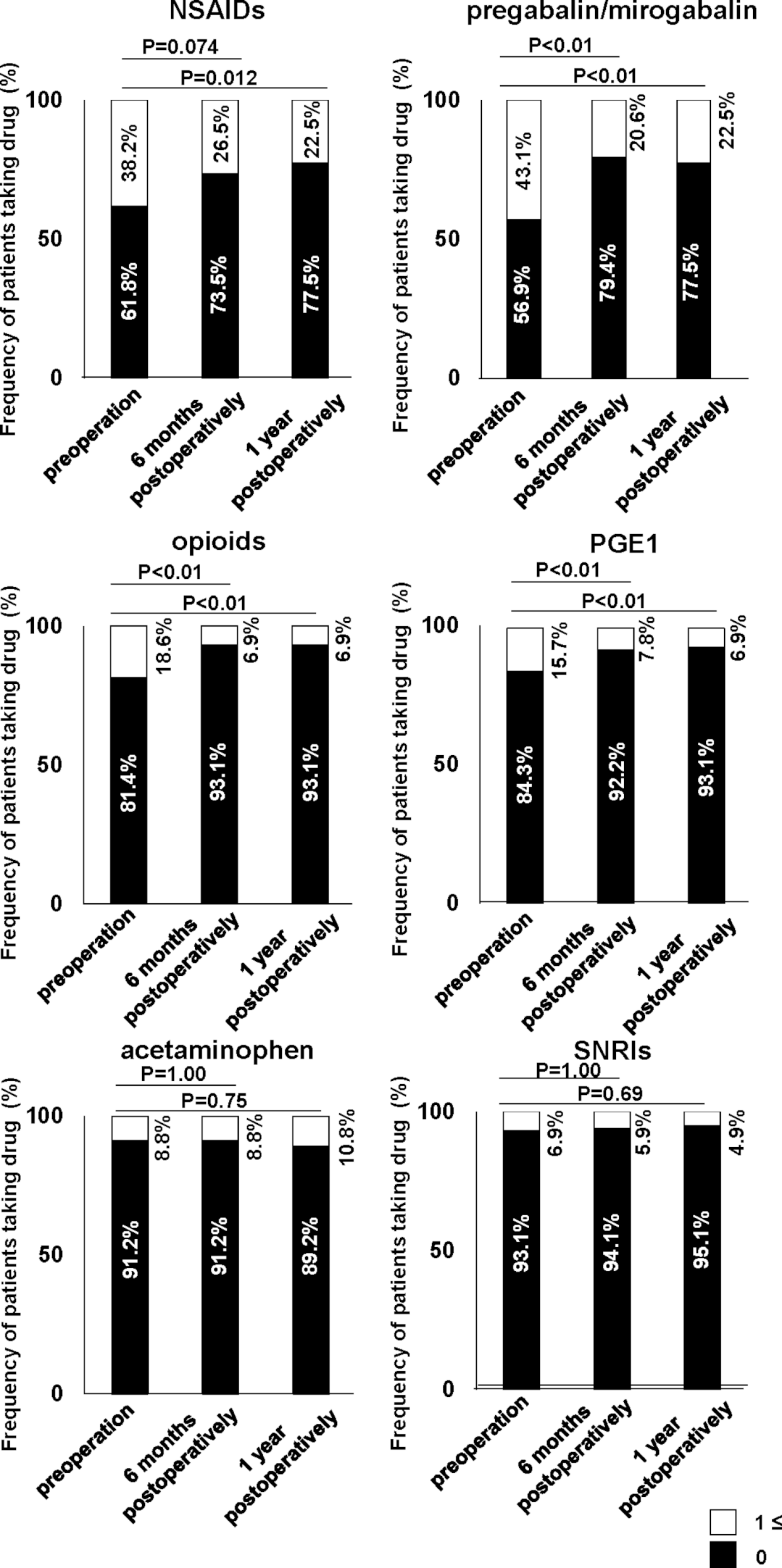
Comparison of the preoperative and 6-month and 1-year postoperative proportion of patients taking NSAIDs, pregabalin/mirogabalin, opioids, SNRIs, PGE1, and acetaminophen

**Fig. 5:**
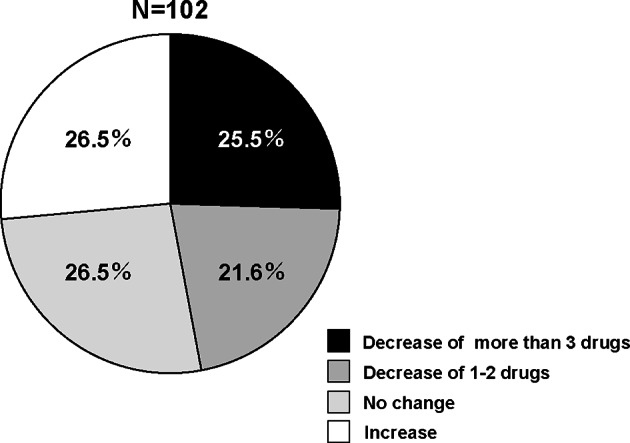
The distribution of changes in the number of drugs 1 year after lumbar spinal surgery

**Fig. 6:**
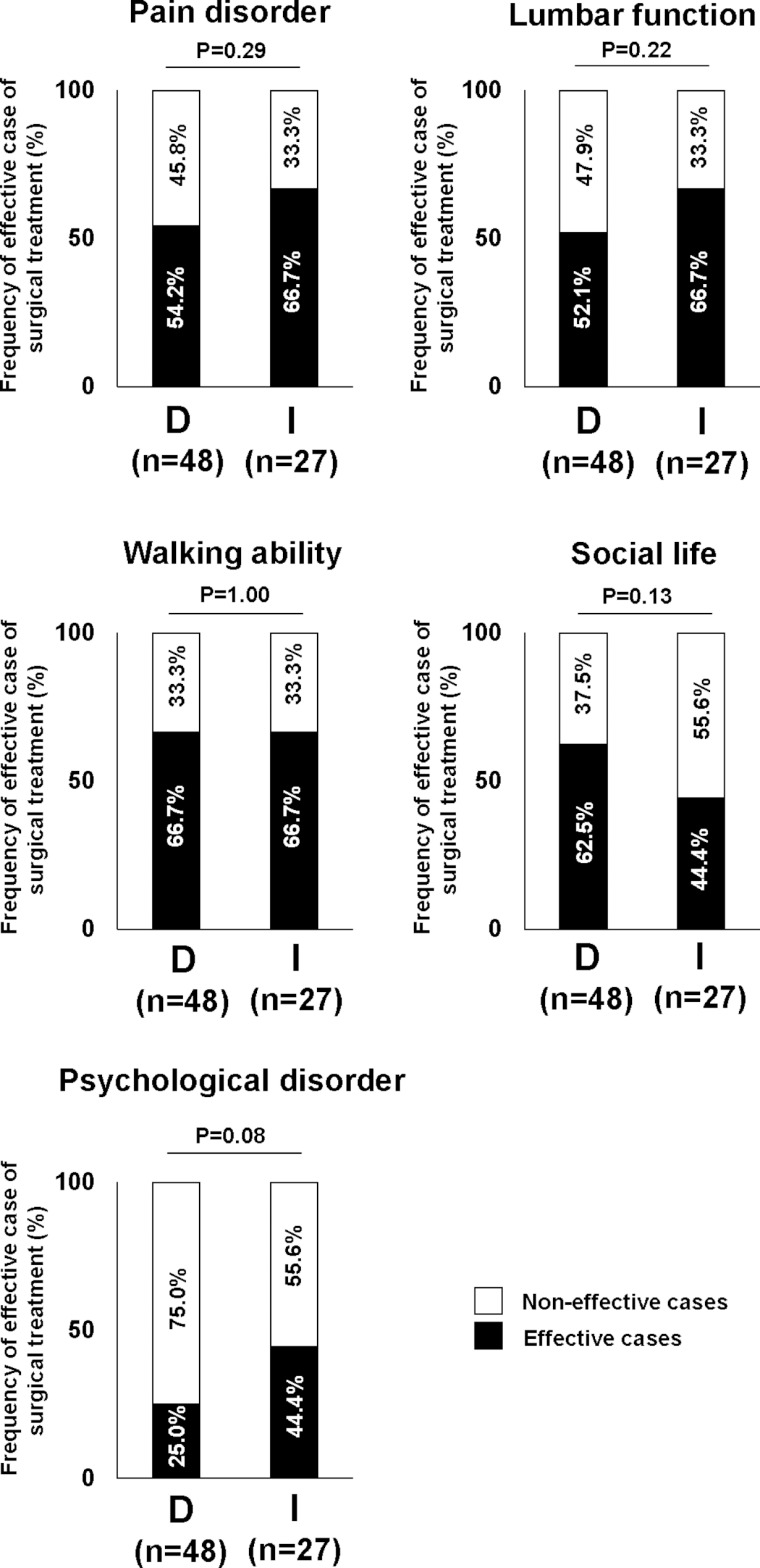
Comparison of the frequency of effective cases of surgical treatment in each domain of JOABPEQ between cases with an increase (I group; n = 27) and cases with a decrease (D group; n = 48) in drugs one year after surgery

Changes in the number of drugs before and after surgery were investigated in 9 categories. The number of drugs for pain relief and digestive diseases at both 6 months and 1 year after surgery was significantly lower than that at preoperation (Fig. 3). The other categories showed almost no change in the number of drugs (Fig. 3). Among the common prescribed drugs for LSS patients, we investigated changes in the proportion of patients taking NSAIDs, pregabalin/mirogabalin, opioids, SNRIs, PGE1, and acetaminophen before and after surgery (Fig. 4). In NSAIDs, the proportion of patients taking them at 1 year after surgery were significantly lower than that before surgery (Fig. 4). In pregabalin/mirogabalin, opioids, and PGE1, the proportion of patients taking them at both 6 months and 1 year after surgery were significantly lower than that before surgery (Fig. 4). Figure 5 showed the distribution of changes in the number of drugs 1 year after surgery. Of the participants, 25.5% had a decrease of more than 3 drugs, 21.6% had a decrease of 1–2 drugs, and 26.5% had no change. In contrast, 26.5% showed an increase in drug consumption. To clarify the relationship between surgical efficacy and postoperative changes in prescription drug counts, we compared the frequency of effective cases of surgical treatment in each domain of JOABPEQ between cases with an increase (I group; n = 27) and cases with a decrease (D group; n = 48) in drugs one year after surgery (Fig. 6). However, they showed no significant differences between two groups (Fig. 6). Next, because Fig. 5 showed that the participants were roughly divided into four equal parts in the distribution of changes in the number of drugs, we focused on the top 1/4 which indicated 3 or more drug reductions after 1-year of surgery. Here, we used a Poisson regression model to identify the factors associated with 3 or more drug reductions after 1-year of surgery. In this analysis, the scores of JOABPEQ were categorized in tertiles because there is no clinical cutoff value for categorizing the scores in each domain of JOABPEQ. In addition, because scores of JOABEPQ have misclassification, we divided them into groups with similar scores. The multi-variable analysis revealed that a higher score in the psychological disorder in JOABPEQ was associated with 3 or more drug reduction after 1-year of surgery (RR, 2.5; 95% CI: 1.0–6.1) (Table 3).

**Table 3:** Poisson regression model of decrease of 3 or more drugs

			Number of patients	Number of decrease of 3 or more drugs	Prevalence of decrease of 3 or more drugs (%)	Multi-variable model*		
						Relative risk (RR)	95% confidence interval	p-value
Age		65–74	47	14	29.8	Reference		
		74-	55	12	21.8	0.9	0.5–1.9	0.87
Sex		Men	56	15	26.8	Reference		
		Women	46	11	23.9	1.0	0.4–2.3	0.99
Metabolic components		No	20	8	40.0	Reference		
		Yes	82	18	22.0	0.6	0.3–1.2	0.13
FBSS		No	94	24	25.5	Reference		
		Yes	8	2	25.0	0.8	0.3–2.3	0.72
Surgical procedure		decompression	59	13	22.0	Reference		
		decompression + fusion	43	13	30.2	1.5	0.7-3.0	0.31
JOABPEQ	Pain disorder	Tertile1 (< 15)	38	6	15.8	Reference		
		Tertile2 (15–57)	32	10	31.3	1.6	0.6–4.3	0.32
		Tertile3 (> 57)	32	10	31.3	1.8	0.6–4.8	0.26
	Lumbar function	Tertile1 (< 26)	35	9	25.7	Reference		
		Tertile2 (26–67)	32	7	21.9	0.5	0.2–1.3	0.16
		Tertile3 (> 67)	35	10	28.6	0.5	0.2–1.4	0.17
	Walking ability	Tertile1 (0)	26	5	19.2	Reference		
		Tertile2 (1–29)	47	12	25.5	1.7	0.7–4.3	0.24
		Tertile3 (> 29)	29	9	31.0	2.4	0.7–8.2	0.15
	Social life	Tertile1 (< 25)	34	10	29.4	Reference		
		Tertile2 (25–49)	35	6	17.1	0.5	0.2–1.4	0.19
		Tertile3 (> 49)	33	10	30.3	0.5	0.2–1.5	0.25
	Psychological disorder	Tertile1 (< 36)	33	6	18.2	Reference		
		Tertile2 (36–50)	36	8	22.2	1.8	0.7–4.5	0.25
		Tertile3 (> 50)	33	12	36.4	2.6	1.0-6.5	0.045

FBSS, Failed back surgery syndrome; JOABPEQ, JOA Back Pain Evaluation Questionnaire

*All variables were included in this table

## Discussion

For the first time, this study followed the prescribed drugs for LSS patients with lumbar spinal surgery. The frequency of polypharmacy in preoperative LSS patients was around two-thirds when the cutoff number of drugs was 6. In addition, lumbar spinal surgery significntly reduced the proportions of polypharmacy of the participants. Finally, our data indicated that psychological conditions might contribute to the postoperative reduction of prescribed drugs for LSS patients who underwent lumbar surgery.

It has been reported that the ADEs, which is higher in the elderly than in the young, occurs over 10% per year [23]. Since polypharmacy causes ADEs associated with drug interactions and increases the incidence of missed or mistaken drug doses, sufficient attention should be paid to polypharmacy among elderlies [11]. The elderly with degenerative musculoskeletal disorders are more likely to be diagnosed with polypharmacy because of chronic musculoskeletal pain. In a recent study, the polypharmacy prevalence (cutoff value = 6 drugs) was 52% among the elderly patients with elective surgical treatment for degenerative musculoskeletal disorders [17]. Because LSS patients commonly have low back and neuropathic pain and multiple comorbidities [18], polypharmacy may be particularly common in LSS patients. Our study reported a high polypharmacy prevalence among LSS patients (66.7%) who were indicated for surgical treatment; therefore, we should pay special attention to this patient group. Our results showed that 72% of LSS patients had at least 1 pain relief drug, while 17% had 3 or more. For LSS patients who took no pain relief medications before surgery (28%), pain relief medications such as NSAIDs, pregabalin, and opioids may have already been discontinued before surgery because none of them worked. As a pain relief drug, NSAIDs have been reported to be potentially inappropriate medications for elderly patients [24]. Furthermore, NSAIDs is predicted to increase the number of drugs as the patient will require them to prevent gastrointestinal bleeding. Consistently, our results showed that 76% of LSS patients had at least one drug for digestive diseases, while 19% had three or more. For example, the combination of proton pump inhibitors can reduce the risk of upper gastrointestinal bleeding in elderly patients. However, although proton pump inhibitors have not been exactly defined as potentially inappropriate medications, they were reported to increase the risk of Clostridium difficile infections and fractures [24]. Therefore, from the viewpoint of polypharmacy, the use of NSAIDs should be especially discouraged as much as possible.

Our data clearly showed that lumbar spinal surgery significantly reduced the number of prescribed drugs in LSS patients. This is mainly due to a decrease in pain relief drugs and concomitantly digestive disease drugs after surgery. In the present study, we examined the relationship between the surgical outcome of pain disorder and the change in the drug counts, but we could not find a significant relationship between them.These findings indicate that the decline in prescription drugs for LSS patients does not depend solely on surgical pain relief. The most important finding of this study was that lumbar spinal surgery significantly reduced the prevalence of LSS patient polypharmacy. Given the increasing number of patients worldwide with musculoskeletal disorders including LSS [1, 2], lumbar spinal surgery can be beneficial for the elderly LSS patients in terms of preventing ADEs such as reduction of physical function, decreased cognitive function, and high risk of falls. Lumbar spinal surgery has been reported to be effective in pain [25], motor function [26], risk of falling [20], social life [27], psychology [28], and healthy life expectancy [29] for LSS patients. Our results added one more advantage to the surgical treatment. While reducing the polypharmacy of elderly patients by lumbar spinal surgery is socioeconomically meaningful, surgical treatment, on the other hand, also raises medical expenses. Therefore, in the future, it is mandatory to make long-term health economics comparisons between LSS patients who did not undergo surgery and those who did.

This study showed that among LSS patients with surgery, some had a decrease of three or more prescribed drugs after surgery, while others had an increase. We performed a multi-variable analysis to determine whether preoperative factors could predict the difference. Although the number of cases was limited and not perfect for the analysis, our results revealed that patients with a reduction of three or more drugs after surgery had relatively good psychological conditions. Considering that previous literature reported that mental health status is one of the risk factors for polypharmacy [30], the number of prescribed drugs in LSS patients with good psychological conditions may be more susceptible to the effects of surgical pain relief. Therefore, our results indicated that taking care of the psychological condition of LSS patients may facilitate the improvement of polypharmacy with lumbar spinal surgery. To increase the efficacy of lumbar spinal surgery on polypharmacy, further identification of associated factors other than psychological condition should be analyzed with larger sample size.

This study has several limitations. First, data for this study were collected from a limited number of patients in a single institution. Especially, a larger sample is needed for a comprehensive multi-variable analysis. Second, more than 20% of initially enrolled patients dropped out during the follow-up period. In general, patients who dropped out tend to have poor postoperative outcomes [31], so our data may be better as an estimate than a true result. Third, the follow-up duration of 1 year was not sufficient to assess the efficacy of lumbar spinal surgery on polypharmacy because some patients have symptoms that change more than one year after surgery. Lastly, the present study could include patients who were prescribed under appropriate decisions and management and those who were not. That is, this study could examine patients who were also diagnosed with polypharmacy but had different implications. To the best of our knowledge, however, this is the first follow-up study to assess the polypharmacy among LSS patients. This study provides useful information for LSS patients and health providers, including doctors and pharmacists.

## Conclusion

This study demonstrated that the prevalence of polypharmacy was high among elderly patients with lumbar spinal surgery for LSS. In addition, our data showed that lumbar spinal surgery was effective for the reduction of polypharmacy in LSS patients. Finally, the multi-variable analysis indicated that better psychological condition was associated with the reduction of prescribed drugs after lumbar spinal surgery.

## Data Availability

The datasets generated and/or analysed during the current study are not publicly available due to limitations of ethical approval involving the patient data and anonymity but are available from the corresponding author on reasonable request.

## Abbreviations

LSS: lumbar spinal canal stenosis
NSAIDs: Non-Steroidal Anti-Inflammatory Drugs
SNRIs: serotonin-noradrenalin reuptake inhibitors
PGE1: prostaglandin E1 analogs
ADEs: adverse drug events
FBSS: failed back surgery syndrome
RDQ: Roland Morris Disability Questionnaire
ZCQ: Zurich Claudication Questionnaire
JOABPEQ: Japanese Orthopaedic Association Back Pain Evaluation Questionnaire
BMI: body mass index
DM: diabetes mellitus
RR: relative risk
CIs: confidence intervals
